# Mitochondrial Calcium Uniporter (MCU)-Mediated Calcium Overload in Psychoactive Drug Neurotoxicity: From Pathogenesis to Therapeutic Targets

**DOI:** 10.3390/ijms26104732

**Published:** 2025-05-15

**Authors:** Xinyan Yang, Yinyu Chen, Gaolin Zheng, Qianyun Nie, Peng Zhang

**Affiliations:** 1 Key Laboratory of Tropical Translational Medicine of Ministry of Education, Department of Forensic Medicine, Hainan Provincial Tropical Forensic Engineering Research Center, Hainan Medical University, Haikou 571199, China; 15338917291@163.com (X.Y.); chenyinyu0728@126.com (Y.C.); 15348893351@163.com (G.Z.); 2 Department of Pathology, School of Basic Medicine and Life Sciences, Hainan Medical University, Haikou 571199, China

**Keywords:** mitochondrial calcium uniporter (MCU), calcium overload, psychoactive substances, neurotoxicity, review

## Abstract

With rapid societal changes and increasing stress levels, the abuse of psychoactive substances has emerged as a global health crisis. Studies indicate that the mitochondrial calcium uniporter (MCU) plays a pivotal role in neurotoxic damage induced by psychoactive substances. As the primary channel for mitochondrial Ca^2+^ uptake, MCU dysfunction can lead to Ca^2+^ overload, oxidative stress, and apoptosis, representing a crucial mechanism underlying neurotoxic damage. Psychoactive substances such as 3,4-Methylenedioxymethamphetamine (MDMA), cocaine, and morphine influence MCU function through multiple pathways, resulting in excessive Ca^2+^ accumulation and mitochondrial dysfunction, ultimately leading to neuronal injury. Although MCU inhibitors have demonstrated potential in alleviating Ca^2+^ overload and improving neural function in preliminary studies, their selectivity and long-term safety require further evaluation. Future research should explore the precise regulatory mechanisms of MCU in neurotoxic damage induced by psychoactive substances and develop more effective targeted therapeutic strategies.

## 1. Introduction

With rapid societal changes and increasing stress levels, millions of people worldwide suffer health damage or even fatal consequences due to psychoactive substance abuse each year. These substances pose a significant public health threat due to their diverse chemical structures and regulatory lag. The abuse of psychoactive substances not only directly endangers individual health—manifesting as addiction, brain dysfunction, and psychological disorders—but also contributes to rising crime rates, family breakdowns, and increased socioeconomic burdens. Therefore, elucidating the mechanisms underlying neurotoxic damage caused by psychoactive substances and identifying effective therapeutic interventions has become an urgent research priority.

In recent years, mitochondrial calcium uniporter (MCU), an essential membrane protein, has garnered increasing research interest [1]. Emerging studies have demonstrated abnormal MCU expression in the mitochondrial inner membrane of individuals with psychoactive substance abuse, primarily involving mitochondrial Ca^2+^ regulation and intracellular signaling pathways. Additionally, Liu Z et al. reported that specific MCU inhibitors could mitigate neurotoxic damage by suppressing MCU activity [2]. These findings suggest that MCU holds promise as a potential therapeutic target for neurotoxic damage induced by psychoactive substances. This review systematically discusses the structure, function, regulatory mechanisms, agonists, and inhibitors of MCU, and its role in the neurotoxic damage induced by psychoactive substances. The aim is to provide insights into its mechanistic implications while offering references for developing more effective targeted therapies for individuals suffering from psychoactive substance abuse.

## 2. Structure and Function of MCU

### 2.1. Structure of MCU

MCU, also known as CCDC109A1, consists of 351 amino acids with a molecular weight of approximately 40 kDa [3]. It is present in most eukaryotes, including protozoa, plants, amoebae, fungi, and metazoans. In 2004, researchers first recorded calcium currents from the inner mitochondrial membrane (IMM) using the patch-clamp technique in isolated mitotic cells [4]. Subsequent studies confirmed that mitochondria are capable of Ca^2+^ uptake due to the presence of an inwardly rectifying, highly selective Ca^2+^ channel in the IMM, a process dependent on the MCU complex [5].

**Figure 1:**
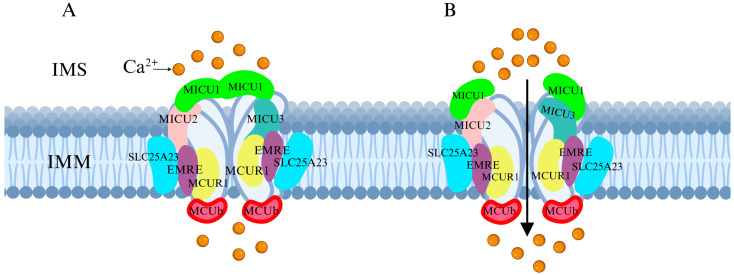
Schematic representation of the MCU complex structure. (**A**) The MCU complex is located in the IMM and consists of MICU family proteins (MICU1, MICU2, and MICU3), the dominant-negative subunit beta of MCU (MCUb), MCUR1, EMRE, and SLC25A23. These components collectively regulate the Ca^2+^ uptake capacity of MCU. (**B**) When Ca^2+^ concentration in the IMS increases, MICU family proteins sense the change and facilitate MCU channel opening, allowing Ca^2+^ influx from the IMS into the IMM.

Recent research has revealed that the MCU channel exists as a heterogeneous protein complex with a molecular mass ranging between 450 and 800 kDa. This complex is structurally intricate, comprising multiple mitochondrial calcium uptake proteins (MICU1, MICU2, and MICU3), a dominant-negative subunit beta of MCU (MCUb), mitochondrial calcium uniporter regulator 1 (MCUR1), essential MCU regulator (EMRE), and solute carrier family 25 member 23 (SLC25A23) (Figure 1A) [6]. These proteins collectively regulate MCU function and control Ca^2+^ transport. MCU itself contains two coiled-coil domains (CC1 and CC2) and two transmembrane domains (TM1 and TM2), both embedded within the IMM. The N- and C-terminal regions of MCU are oriented toward the mitochondrial matrix, connected by a highly conserved short loop containing the DIME motif (Asp-Ile-Met-Glu). This sequence is essential for Ca^2+^ permeability, as mutations in its negatively charged residues significantly impact MCU function [2,6,7].

The MICU family proteins contain EF-hand domains, which function as Ca^2+^-binding sites, allowing them to sense Ca^2+^ concentration fluctuations in the cytosol or intermembrane space (IMS), thereby regulating MCU channel activity and mitochondrial Ca^2+^ uptake (Figure 1B) [8,9]. Additionally, other regulatory proteins modulate MCU activity through various mechanisms, influencing mitochondrial Ca^2+^ uptake capacity.

### 2.2. Function of MCU

Mitochondria play essential roles in cellular physiology, including energy production, metabolic regulation, intracellular Ca^2+^ homeostasis, and apoptotic signaling [10]. As a crucial second messenger, Ca^2+^ regulates numerous cellular activities, including transmission of neuronal signal and muscle contraction. MCU-mediated Ca^2+^ uptake into mitochondria via the IMM is vital for these physiological processes [11].

**Figure 2:**
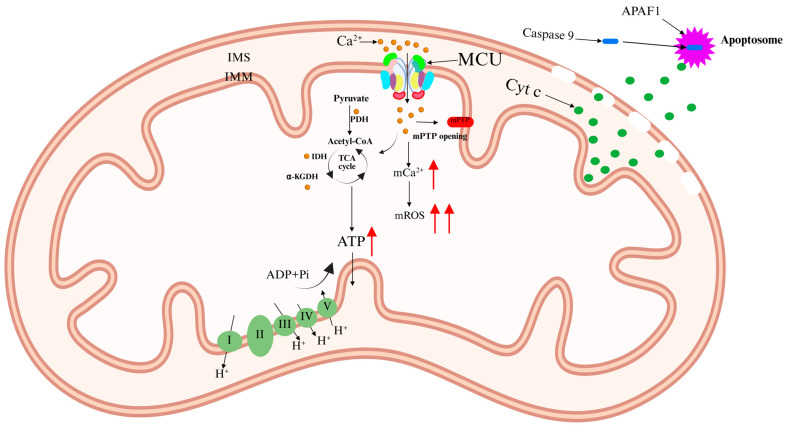
Regulatory effects of excessive Ca^2+^ on MCU. Ca^2+^ influx through MCU in the IMM activates PDH, IDH, and α-KGDH, regulating the TCA cycle, increasing electron flux through the respiratory chain, and promoting ATP production. However, excessive Ca^2+^ accumulation in the IMM induces mROS generation, mitochondrial membrane depolarization, and subsequent opening of the mPTP. This leads to Cyt c release into the cytoplasm, where it binds to caspase-9 and APAF1 to form the apoptosome, triggering a proteolytic cascade and ultimately resulting in apoptosis. The red arrow represents an increase.

Ca^2+^ activates three Ca^2+^-sensitive dehydrogenases within the mitochondrial matrix: pyruvate dehydrogenase (PDH), isocitrate dehydrogenase (IDH), and alpha-ketoglutarate dehydrogenase (α-KGDH) [12,13]. This activation enhances the tricarboxylic acid (TCA) cycle, increasing electron flux through the respiratory chain and boosting ATP production. However, excessive Ca^2+^ accumulation in the mitochondrial matrix triggers detrimental effects, including elevated mitochondrial reactive oxygen species (mROS) levels and mitochondrial membrane depolarization. This condition leads to the opening of the mitochondrial permeability transition pore (mPTP), resulting in mitochondrial swelling and the release of cytochrome c (Cyt c). Cyt c interacts with apoptotic peptidase activator 1 (APAF1) to form the apoptosome [14]. This complex recruits and activates caspase-9, initiating a proteolytic cascade that ultimately leads to apoptosis (Figure 2) [11,15]. Notably, apoptosis is a key mechanism underlying neurotoxicity induced by psychoactive substances [16].

MCU dysfunction is closely associated with various diseases. In cardiovascular disorders, reduced MCU expression contributes to Ca^2+^ dysregulation, whereas MCU overexpression has been found to exert protective effects by enhancing cardiovascular cell function [17,18]. In cancer, MCU is often upregulated, as observed in breast and pancreatic cancers [19,20], and suppressing MCU expression has been shown to inhibit tumor growth and metastasis. In the nervous system, neurons are highly sensitive to Ca^2+^ signaling perturbations, which can contribute to neurodegenerative diseases such as Alzheimer’s disease and Parkinson’s disease [21]. Studies have demonstrated that excessive MCU activity leads to mitochondrial Ca^2+^ overload, exacerbating neurotoxicity and excitotoxicity [22,23].

## 3. Regulatory Mechanisms of MCU

The regulation of MCU involves multiple levels of control, including protein–protein interactions, fluctuations in Ca^2+^ concentration, and the influence of various regulatory factors. In particular, under the influence of psychoactive substances, MCU function may be significantly disrupted, leading to excessive mitochondrial Ca^2+^ accumulation and subsequent neurotoxic effects. Therefore, elucidating the precise regulatory mechanisms of MCU, especially its role in neurotoxicity, may provide new insights and therapeutic targets for neurodegenerative diseases and neurotoxicity induced by psychoactive substance abuse.

### 3.1. Regulation of MCU by Associated Proteins

**Figure 3:**
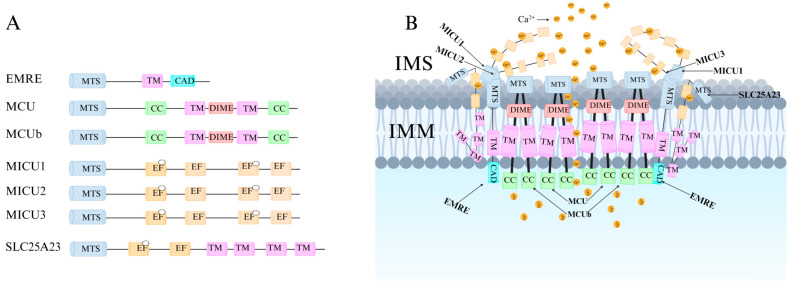
MCU: composition and regulation. (**A**) Linear domain maps of the uniporter subunits, showing each protein’s predicted mitochondrial targeting signal (MTS), transmembrane helix (TM), EF-hand motif, calcium-binding sites (white circles), conserved DIME motif, coiled-coil domain (CC), and C-terminal acidic domain (CAD). (**B**) MCU channel activity is controlled by a multilayered network of subunits: MICU1/2/3 sense cytosolic Ca^2+^ via EF-hands and gate the pore; MCUb serves as a negative regulator; MCUR1 stabilizes the MCU–EMRE interface and links the complex to the mPTP; EMRE activates the channel and anchors it to specific inner-membrane regions; and SLC25A23, through its EF-hands, heightens mitochondrial Ca^2+^ sensitivity.

MCU plays a crucial role in maintaining cellular homeostasis by regulating metabolism, energy production, signal transduction, and cell death. The Ca^2+^ uptake function of MCU is finely regulated by the MICU family, including MICU1, MICU2, and MICU3. These proteins contain EF-hand domains that allow them to sense fluctuations in cytosolic Ca^2+^ concentrations. When cytosolic Ca^2+^ levels reach a certain threshold, MICU1 and MICU2 form a heterodimer, which binds Ca^2+^ at the EF-hand domains, inducing a conformational change that leads to their dissociation from the MCU complex. This process regulates MCU activity and participates in cell death regulation (Figure 3A) [24,25,26,27].

MICU1 and MICU2 are widely expressed in most tissues, whereas MICU3 is particularly abundant in neurons. MICU3 enhances MCU sensitivity to Ca^2+^, facilitating Ca^2+^ uptake into mitochondria within axons, thereby accelerating ATP synthesis and maintaining the complex metabolic functions of neuronal cells [28]. Additionally, MCUb acts as a dominant-negative regulatory subunit of MCU. Structurally similar to MCU, excessive MCUb expression replaces MICU1 and MICU2 within the functional MCU complex, reducing mitochondrial Ca^2+^ uptake capacity [29].

The precise function of MCUR1 remains unclear. Some studies suggest that MCUR1 influences mitochondrial Ca^2+^ uptake by modulating interactions between MCU and EMRE [30]. As an essential subunit of MCU, MCUR1 interacts with both MCU and EMRE, and its absence results in an incomplete MCU complex, thereby inhibiting mitochondrial Ca^2+^ uptake. Research by Paupe V et al. indicates that MCUR1 also regulates the threshold for mPTP opening by modulating interactions between MCU and the mPTP complex [31]. However, other studies suggest that MCUR1 is not a direct regulator of MCU but instead functions as an assembly factor for Cyt c oxidase [32]. Overall, MCUR1 serves as a critical bridge connecting MCU and mPTP.

EMRE is a single-pass transmembrane protein specific to multicellular organisms and is an essential regulatory factor of the MCU complex. When MCU is expressed alone, it does not exhibit uniporter activity; only when co-expressed with EMRE does MCU become functional [33]. EMRE precisely localizes MCU to specific regions of the IMM rather than to the cristae invaginations, which may play a vital role in mitochondrial Ca^2+^ uptake regulation [34]. Studies indicate that the MCU-EMRE complex can dimerize [35,36], though this dimerization is not essential for channel function. Instead, it may facilitate the spatial distribution of the uniporter at contact sites between the inner and outer mitochondrial membranes, enhancing its responsiveness to intracellular Ca^2+^ signals and promoting efficient Ca^2+^ transfer from the endoplasmic reticulum to the mitochondrial matrix, leading to increased mitochondrial Ca^2+^ levels [33,34,37].

SLC25A23, a member of the solute carrier family, is a multi-pass transmembrane protein with three EF-hand domains [38,39]. This structure confers high Ca^2+^ sensitivity, and its Mg^2+^-ATP/Pi transporter function is similar to other Ca^2+^-activated channels and transporters. Although SLC25A23 can modulate mitochondrial Ca^2+^ influx, it does not affect the rate or total amount of mitochondrial Ca^2+^ efflux.

Previous studies have demonstrated that downregulation of MCU significantly reduces mitochondrial Ca^2+^ uptake, both in live cells treated with Ca^2+^-mobilizing agonists and in permeabilized cells subjected to Ca^2+^ buffering perfusion. However, this alteration does not affect the fundamental properties of mitochondria (Figure 3B) [6,40].

### 3.2. Other Regulatory Mechanisms of MCU

#### 3.2.1. Regulation via the CaMKII-CREB Signaling Pathway

**Figure 4:**
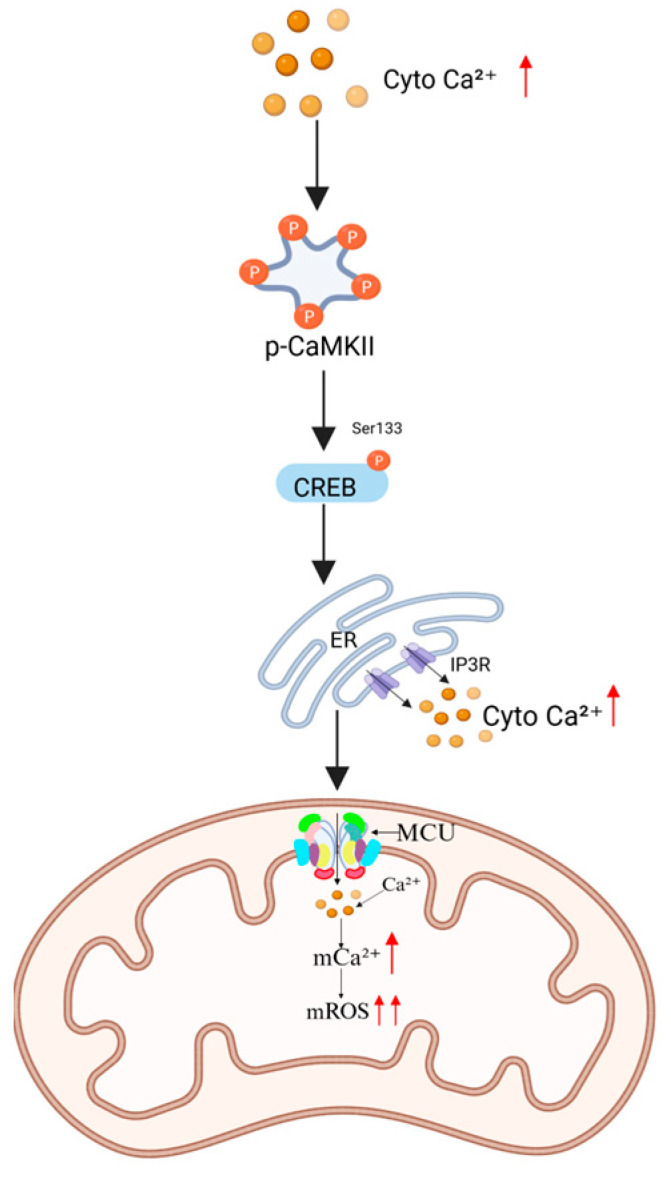
Regulatory mechanism of the CaMKII-CREB signaling pathway. Increased cytoplasm Ca^2+^ (cyto Ca^2+^) activates CaMKII, inducing phosphorylation of CREB at Ser133. This enhances ER IP3R expression, resulting in cytosolic Ca^2+^ overload. Subsequent MCU-mediated mitochondrial Ca^2+^ uptake aggravates oxidative stress. The red arrow represents increase/upregulation.

When intracellular Ca^2+^ concentrations increase, calmodulin-dependent kinase II (CaMKII) undergoes autophosphorylation and activation [41]. This activation promotes the phosphorylation of cAMP response element-binding protein (CREB) at Ser133, enhancing its DNA-binding affinity and subsequently upregulating the expression of endoplasmic reticulum (ER) Ca^2+^ release channels, such as inositol 1,4,5-triphosphate receptors (IP3Rs). Excessive Ca^2+^ release from ER stores leads to cytosolic Ca^2+^ overload, which in turn drives mitochondrial Ca^2+^ uptake Via an MCU-dependent mechanism, exacerbating mitochondrial oxidative stress (Figure 4) [42].

#### 3.2.2. Kinase-Mediated Post-Translational Modifications of MCU

Proline-rich tyrosine kinase 2 (Pyk2) is activated under oxidative stress conditions and phosphorylates MCU at Tyr107, promoting MCU monomer oligomerization into functional calcium channels. This modification significantly enhances mitochondrial Ca^2+^ uptake capacity [43]. Under conditions of cellular energy deficiency, AMP-activated protein kinase (AMPK) phosphorylates MCU at Ser57, inducing a conformational change that facilitates mitochondrial Ca^2+^ influx. This process activates the pyruvate dehydrogenase complex, accelerating acetyl-CoA production to sustain ATP supply [44].

#### 3.2.3. MicroRNA-Mediated Post-Transcriptional Regulatory Network

MicroRNAs (miRNAs) are critical post-transcriptional regulators involved in numerous physiological processes, including cell proliferation, differentiation, metabolism, and apoptosis. Recent studies have demonstrated that multiple miRNAs can directly target MCU mRNA, modulating its translation and thereby influencing mitochondrial Ca^2+^ uptake and related signaling pathways. For instance, miR-129-3p, miR-1, miR-25, miR-138-5p, and miR-340 have been shown to selectively bind to MCU mRNA, suppressing its translation and reducing mitochondrial Ca^2+^ uptake capacity [45,46]. This regulatory mechanism plays a crucial role under various physiological and pathological conditions, including excitotoxicity in neurons, ischemia–reperfusion injury, tumorigenesis, and drug resistance.

## 4. Specific Agonists and Inhibitors of MCU

### 4.1. MCU-Specific Agonists and Their Mechanisms of Action

**Figure 5:**
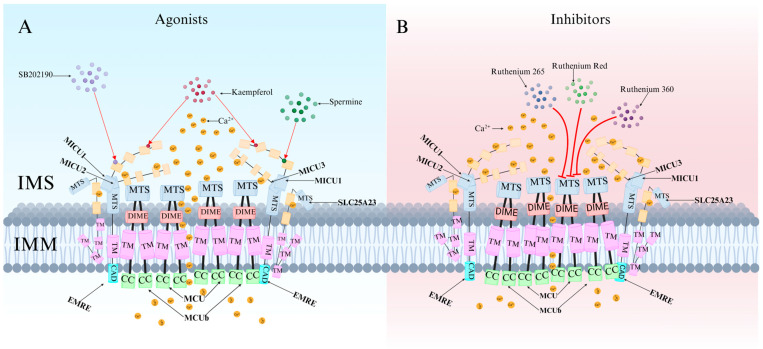
Mechanism of MCU regulation by specific agonists and inhibitors. (**A**) Specific activators (spermine, kaempferol, SB202190) bind the EF-hand of MICU1, disable its gatekeeping function, and thereby enhance mitochondrial Ca^2+^ uptake (arrows denote activation). (**B**) Specific inhibitors target the MCU complex (flat-headed bars denote inhibition).

Currently known MCU-specific agonists include spermine, kaempferol, and SB202190. These compounds exert their effects by modulating MICU1, potentially by binding to the EF-hand domain of MICU1 and blocking its gatekeeping activity, thereby enhancing mitochondrial Ca^2+^ uptake (Figure 5A) [3,30].

### 4.2. Classification and Characteristics of MCU-Specific Inhibitors

MCU-specific inhibitors include ruthenium red (RuRed), ruthenium 360 (Ru360), ruthenium 265 (Ru265), mitoxantrone (MTX), DS16570511, metoprolol, and KN-93. Among them, RuRed is the most commonly used MCU-specific inhibitor. It blocks MCU channels to reduce Ca^2+^ and Fe^2+^ accumulation, prevents mitochondrial depolarization, alters synaptic activity, improves mitochondrial function, and decreases DNA damage and apoptosis [47,48]. However, due to challenges in RuRed purification, commercially available RuRed formulations are not pure compounds but mixtures of several ruthenium-amine complexes [49].

Ru360 is the primary active component of RuRed mixtures. It selectively inhibits MCU without affecting Na^+^/Ca^2+^ channels or L-type Ca^2+^ channels and does not significantly impact the cell cycle, proliferation, or viability [50]. Studies have shown that Ru360 prevents glutamate-induced excitotoxicity in cortical neurons while mitigating oxidative stress in microglia and inhibiting amyloid-beta (Aβ)-induced apoptosis [51,52]. Compared to Ru360, Ru265 offers greater selectivity and cellular permeability, with over twice the cellular uptake efficiency of Ru360 [8]. Furthermore, Ru265 exhibits lower toxicity than many ruthenium-based compounds and provides more effective MCU inhibition than Ru360.

Organic molecules such as MTX and DS16570511 lack MCU-specific selectivity, often producing off-target biological effects [53,54]. DS16570511 inhibits both MCU and MICU1, alleviating mitochondrial Ca^2+^ overload while increasing cytosolic Ca^2+^ concentration and muscle contraction frequency.

KN-93 effectively attenuates bupivacaine-induced neurotoxicity by inhibiting the CaMKIIα-MCU signaling pathway [2]. Additionally, two small-molecule compounds, MCU-i4 and MCU-i11, have been identified through high-throughput screening. These compounds reduce mitochondrial Ca^2+^ influx by directly binding to MICU1-specific sites (Figure 5B). Notably, their inhibitory effects are observed only in cellular contexts [55].

## 5. Research on MCU in Neurotoxic Damage Induced by Psychoactive Substances

Psychoactive substances refer to compounds that alter brain function, influencing cognition, emotions, consciousness, behavior, or perception. Recent studies have revealed that psychoactive substances can disrupt MCU function, leading to neuronal Ca^2+^ overload and mitochondrial dysfunction, thereby inducing oxidative stress, apoptosis, and neurodegeneration. Understanding the role of MCU in these processes not only provides insights into the mechanisms of neurotoxic damage caused by psychoactive substances but also offers potential directions for drug interventions and neuroprotection. This section systematically examines the regulatory effects of different psychoactive substances on MCU and their associated neurotoxic damage, providing a theoretical foundation for targeted therapeutic strategies (Table 1).

### 5.1. 3,4-Methylenedioxymethamphetamine (MDMA)

**Figure 6:**
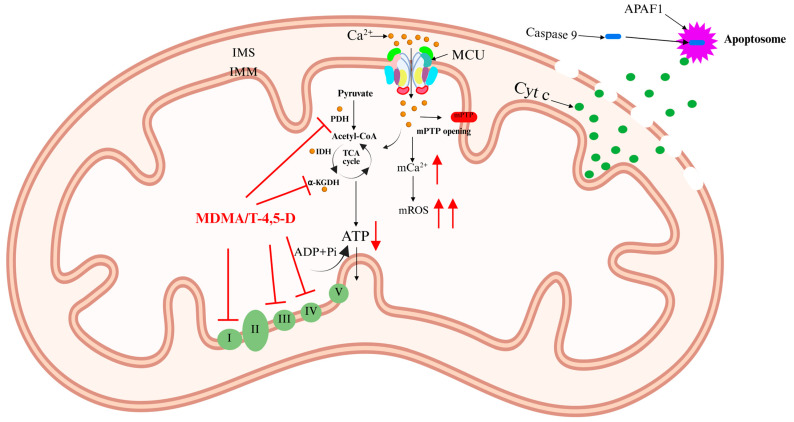
Mechanism of MDMA and its metabolite T-4,5-D in MCU-mediated neurotoxicity. MDMA targets and inhibits mitochondrial complexes I and III, leading to mitochondrial Ca^2+^ overload, increased ROS production, and oxidative stress. Additionally, the MDMA metabolite T-4,5-D inhibits PDH and α-KGDH, while also inactivating mitochondrial complexes I and IV, further exacerbating mitochondrial membrane depolarization, ATP depletion, and Ca^2+^ overload, ultimately leading to neuronal apoptosis. The red arrow represents material change, with an upward direction indicating an increase and a downward direction indicating a decrease; The red flat-headed bars indicates inhibition.

MDMA, commonly known as “ecstasy”, is a widely abused novel psychoactive substance with both stimulant and hallucinogenic properties. MDMA is frequently misused in nightlife and party settings. A single low-dose administration of MDMA produces effects such as euphoria, wakefulness, relaxation, enhanced sociability, and increased emotional connection with others [56]. However, animal studies have shown that chronic MDMA abuse leads to central nervous system (CNS) damage, particularly neurotoxicity, which may progressively manifest over time [57]. Mitochondrial dysfunction is one of the key mechanisms underlying MDMA-induced neurotoxic damage. Disrupted Ca^2+^ homeostasis and ATP depletion play crucial roles in MDMA-induced neurotoxicity. Previous studies have reported that MDMA inhibits mitochondrial complexes I and III, significantly increasing ROS levels and inducing oxidative stress. This oxidative damage impairs cellular components such as Ca^2+^ pumps, ATPase, and Na^+^-K^+^-ATPase, leading to abnormal intracellular Ca^2+^ accumulation, MCU dysfunction, and ultimately cell death [58,59]. Additionally, MDMA’s metabolite, tryptamine-4,5-dione (T-4,5-D), inhibits PDH and α-KGDH and inactivates mitochondrial complexes I and IV, exacerbating mitochondrial membrane depolarization and ATP depletion [60]. This process indirectly activates MCU, resulting in mitochondrial Ca^2+^ overload and ultimately neuronal apoptosis (Figure 6). However, direct evidence demonstrating the ability of MCU inhibitors to reverse MDMA-induced neurotoxic damage is still lacking. Future studies should explore this hypothesis through gene knockout models or specific inhibitor intervention experiments.

### 5.2. Cocaine

Cocaine, an alkaloid extracted from coca leaves, has potent anesthetic properties, high permeability, and strong CNS stimulant effects, making it one of the most widely abused psychoactive substances. Cocaine acts on the brain’s reward pathways, leading to addiction, and chronic use results in severe CNS dysfunction. Moreover, genetic alterations in cocaine abusers may affect subsequent generations. Astrocytes, a subtype of glial cells in the CNS, regulate synaptic transmission and plasticity through intracellular Ca^2+^ signaling. Recent studies indicate that cocaine induces neurotoxicity by modulating MCU function in astrocytes. In the brain tissue of cocaine abusers, acetyl-CoA expression is elevated in astrocytes [61]. Increased acetyl-CoA activates G protein-coupled receptors (GPCRs), leading to elevated mitochondrial Ca^2+^ levels [62], which enhance MCU-mediated Ca^2+^ uptake, subsequently increasing mROS production. Excessive mROS impairs neuronal health and exacerbates neurotoxic damage. Since mitochondria serve as the primary site for fatty acid oxidation (FAO) and mitochondrial Ca^2+^ is a key regulator of mitochondrial bioenergetics, MCU knockdown has been shown to activate AMPK-dependent lipid metabolic reprogramming, inhibiting FAO while shifting energy metabolism toward anaerobic glycolysis. This metabolic shift maintains neuronal energy supply through lactate production [61]. These findings suggest that cocaine-induced neurotoxicity is closely related to metabolic imbalance driven by excessive MCU activation. Thus, targeting MCU inhibition or AMPK activation may serve as a potential therapeutic strategy to alleviate cocaine addiction and mitigate neurodegeneration.

### 5.3. Morphine

Morphine is a potent opioid widely used for pain management, particularly in postoperative pain and cancer-related pain treatment. However, long-term morphine use leads to adaptive changes in the nervous system, resulting in tolerance and drug dependence. Morphine tolerance necessitates increasing doses to achieve the same analgesic effect, which may exacerbate neuronal damage and neurotoxicity. Studies have shown that morphine tolerance induces spinal neuronal neurotoxicity, a process involving MCU [63]. Recent research has demonstrated that CREB regulates neuroplasticity, contributing to hyperalgesia and chronic pain, and plays a crucial role in synaptic plasticity, learning, and memory [64]. Both short-term and long-term morphine exposure modulates phosphorylated CREB (pCREB) expression across different brain regions, mediating epigenetic regulation of MCU transcription. In vertebrates, the cytoplasmic polyadenylation element binding (CPEB) mRNA protein family consists of four members (CPEB1-4). Among them, CPEB1 is essential for mitochondrial energy production in neurons and stabilizes the activated cytoplasmic polyadenylation-specific factor complex [65]. CPEB1 recruits poly (A) polymerase, catalyzing poly (A) tail elongation, which participates in translational activation. As a result, MCU overexpression enhances mitochondrial Ca^2+^ uptake, leading to mitochondrial Ca^2+^ overload and neurotoxicity. Animal studies have shown that MCU inhibitors such as Ru360, or pharmacological blockers targeting pCREB or CPEB1 upregulation, reduce morphine tolerance-related behavioral responses. These findings suggest that MCU inhibition may help prevent opioid tolerance [66]. Future research should explore epigenetic regulatory mechanisms, such as histone modifications and non-coding RNA involvement in MCU expression, to develop multi-target intervention strategies.

### 5.4. Cannabis

**Figure 7:**
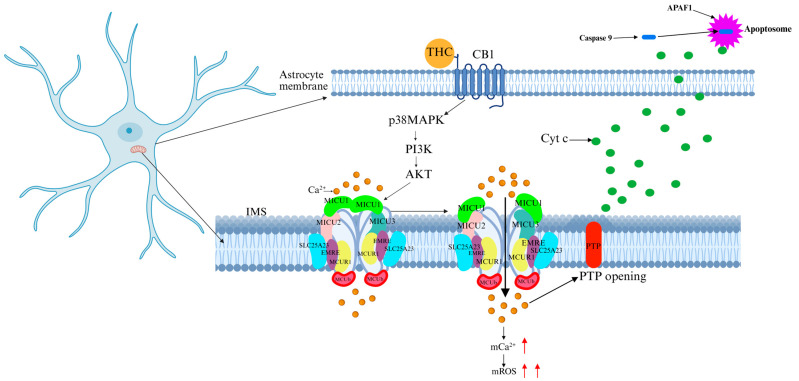
Mechanism of THC-induced neurotoxicity Via MCU. The psychoactive compound THC in cannabis binds to the cannabinoid receptor CB1, activating the PI3K-AKT pathway, which phosphorylates MICU1 on MCU, leading to MCU channel opening and Ca^2+^ influx into the IMM. This results in mitochondrial Ca^2+^ overload, oxidative stress, mPTP opening, Cyt c release, and apoptosis, contributing to THC-induced neurotoxicity. The red arrow represents an increase.

Cannabis is the most widely abused psychoactive substance globally, encompassing the largest variety of compounds and accounting for the highest sales volume. Due to its hallucinogenic and addictive properties, cannabis is classified as a strictly controlled substance in most countries. The primary psychoactive component of cannabis, Δ9-tetrahydrocannabinol (Δ9-THC), exerts potent effects on the CNS. Studies have revealed that THC alters mitochondrial function in brain and muscle tissues [67,68]. By binding to cannabinoid receptors (CB1 and CB2, both G-protein-coupled receptors), THC disrupts neurotransmitter systems in the brain. In particular, CB1 receptor activation induces neurotransmitter imbalances, increases oxidative stress and ROS production, and impairs mitochondrial function [69]. CB1 receptor activation has been shown to promote mitochondrial Ca^2+^ accumulation in both cultured astrocytes and in vivo animal models [70]. CB1 modulates soluble adenylyl cyclase (sAC) activity, which regulates mitochondrial oxygen consumption in neurons and astrocytes [69,71]. Additionally, under chronic or excessive cannabis use, CB1 activation triggers the protein kinase B/AKT pathway, leading to AKT-mediated phosphorylation of MICU1, which increases Ca^2+^ influx into the mitochondrial matrix. This process results in mitochondrial Ca^2+^ overload, mitochondrial membrane potential disruption, and mPTP opening, ultimately causing neuronal apoptosis (Figure 7) [72]. Cannabis abuse is associated with cognitive impairment, mood disturbances, and neurodegeneration. Previous clinical studies have shown that selective CB1 antagonists partially reverse THC-induced neurotoxicity; however, these agents may interfere with the physiological functions of the endocannabinoid system [73]. Therefore, targeting MCU-specific modulators rather than cannabinoid receptor antagonists may provide a safer therapeutic approach.

### 5.5. Methamphetamine (METH)

METH, commonly known as “crystal meth”, is a highly addictive CNS stimulant. While short-term use induces euphoria, heightened alertness, and increased energy, it also leads to aggressiveness, anxiety, and paranoia. Chronic METH use, however, causes severe neurotoxicity, manifesting as brain damage, cognitive and memory impairments, cardiovascular complications, and immune suppression. Due to its high addiction potential and profound effects on CNS function, METH is classified as an illicit substance in most countries. The neurotoxic effects of METH are primarily linked to oxidative stress and mitochondrial dysfunction. Studies have demonstrated that prolonged METH exposure leads to MCU overactivation, resulting in excessive mitochondrial Ca^2+^ accumulation, triggering mitochondrial stress and ROS production [74]. These changes destabilize the mitochondrial membrane, causing alterations in membrane permeability and loss of membrane potential. Ca^2+^ overload, in conjunction with pro-apoptotic protein Bax, induces mPTP opening, facilitating the release of Cyt c and apoptosis-inducing factor (AIF). This process activates both caspase-3-dependent and caspase-3-independent apoptotic pathways, promoting cell death and neurotoxicity [75]. Given that MCU serves as the central regulatory hub for mitochondrial Ca^2+^ influx, its dysfunction plays a critical role in METH-induced mitochondrial-dependent cell death. Thus, targeting MCU may represent a novel therapeutic strategy for mitigating METH-induced neurotoxicity.

### 5.6. Ketamine

Ketamine, commonly known as ‘K’, is a derivative of phencyclidine and acts as a non-competitive N-methyl-D-aspartate receptor (NMDAR) antagonist. It was first synthesized by Calvin Stevens in 1962. Due to its rapid absorption, fast onset, mild respiratory suppression, and limited cardiovascular effects, ketamine was introduced as a human anesthetic in 1970. However, in recent years, ketamine has been misused as a hallucinogen due to its dissociative and euphoric effects, particularly in club and party settings. Several recent studies have demonstrated that ketamine disrupts Ca^2+^ signaling, induces DNA fragmentation, increases ROS production, impairs mitochondrial function, and triggers apoptosis [76,77]. Excessive glutamate release is a major mechanism underlying excitotoxic cell damage. Chronic ketamine abuse leads to compensatory upregulation of the NMDAR NR1 subunit, a process in which persistent or prolonged NMDAR blockade triggers adaptive regulation, making neurons overly responsive to normal extracellular glutamate levels. This phenomenon drives excessive Ca^2+^ influx, elevating intracellular Ca^2+^ concentrations and inducing ROS production Via the CaMKII/CaM/ERK signaling pathway. Subsequently, ROS activates MCU, leading to mitochondrial Ca^2+^ overload, respiratory suppression, and uncoupling of oxidative phosphorylation, ultimately promoting Cyt c release from the mitochondrial membrane into the cytoplasm, triggering apoptosis [78]. Additionally, cytosolic Ca^2+^ stimulates nitric oxide synthase (NOS) to produce NO, which inhibits complex IV, further increasing ROS generation and exacerbating oxidative stress [79]. Animal studies have shown that ketamine abuse induces hyperphosphorylation of Tau protein at Ser202, Ser396, and Trp205, impairing synaptic function in hippocampal neurons and increasing neurotoxicity [80]. However, the precise mechanisms underlying ketamine-induced neurotoxicity and neuronal apoptosis remain unclear. Whether MCU inhibitors can mitigate ketamine-induced neurotoxicity requires further investigation.

## 6. Conclusions and Future Perspectives

This review systematically highlights the crucial role of MCU in neurotoxic damage induced by psychoactive substances. With the worsening global crisis of psychoactive substance abuse, it is imperative to clarify the underlying neurotoxic mechanisms. As the core channel regulating mitochondrial Ca^2+^ uptake in the inner mitochondrial membrane, MCU dysfunction is closely linked to Ca^2+^ homeostasis imbalance, mitochondrial dysfunction, oxidative stress, and apoptosis. Studies have demonstrated that various psychoactive substances—including MDMA, cocaine, morphine, cannabis, METH, and ketamine—disrupt MCU function via different mechanisms, leading to mitochondrial Ca^2+^ overload and neurodegeneration. For instance, MDMA inhibits mitochondrial complex activity, increasing ROS levels, while morphine upregulates MCU expression via the CREB signaling pathway, exacerbating Ca^2+^-dependent neurotoxicity. Additionally, MCU-specific inhibitors (e.g., Ru360, Ru265) have demonstrated potential in alleviating Ca^2+^ overload, restoring mitochondrial function, and exerting neuroprotective effects in in vitro and animal models, providing new directions for drug intervention.

Despite significant progress, further research is required in several key areas. The precise mechanisms by which MCU mediates psychoactive substance-induced neurotoxicity remain unclear, necessitating single-cell sequencing and gene-editing approaches to elucidate these pathways. Given that current MCU inhibitors suffer from low purity and off-target effects, future research should focus on developing highly selective, low-toxicity small molecules or gene therapies for precise MCU regulation and neuroprotection. Furthermore, the functional heterogeneity of MCU across different tissues and diseases, particularly in comorbid models of neurodegenerative diseases and drug addiction, remains to be fully elucidated. Additionally, the long-term inhibition of MCU must be evaluated for its potential impact on physiological Ca^2+^ signaling. Advanced models, such as organoids and non-human primates, should be utilized to assess safety and efficacy before clinical translation. In conclusion, MCU serves as a critical node in psychoactive substance-induced neurotoxicity, making it a promising target for both mechanistic research and therapeutic development. Future studies should integrate multi-omics and interdisciplinary approaches to translate fundamental discoveries into clinical applications.

## Figures and Tables

**Table 1:** Drugs–MCU interaction pathways leading to mitochondrial neurotoxicity.

Drugs	MCU-Related Mechanisms	Final Effects
MDMA	Inhibits mitochondrial complexes I/III → ROS↑, Ca^2+^ overload → ATP depletion → apoptosis. Metabolite T-4,5-D inhibits PDH/α-KGDH and inactivates complexes I/IV.	Mitochondrial Ca^2+^ overload → apoptosis
Cocaine	Astrocyte acetyl-CoA↑ → GPCR activation → ↑MCU-mediated mitochondrial Ca^2+^ uptake → mROS↑→ neurodegeneration. MCU knockdown activates AMPK-dependent glycolysis.	Metabolic imbalance → neurodegeneration
Morphine	Epigenetic MCU upregulation Via pCREB/CPEB1 → mitochondrial Ca^2+^ overload → tolerance-related neurotoxicity. CPEB1 stabilizes polyadenylation complexes.	Morphine tolerance → neuronal damage
Cannabis	CB1 activation → AKT-mediated MICU1 phosphorylation → ↑MCU-driven Ca^2+^ influx → mitochondrial depolarization → mPTP opening → apoptosis.	Mitochondrial Ca^2+^ overload → apoptosis
METH	MCU overactivation → mitochondrial Ca^2+^ overload → Bax activation → mPTP opening → Cyt c/AIF release → caspase-dependent/independent apoptosis.	Mitochondrial stress → caspase-dependent/independent apoptosis
Ketamine	ROS↑ Via CaMKII/CaM/ERK → MCU activation → mitochondrial Ca^2+^ overload → Cyt c release → apoptosis. NMDAR NR1 subunit upregulation → glutamate excitotoxicity → Tau hyperphosphorylation.	Oxidative phosphorylation uncoupling → apoptosis

“↑” Indicates increase/upregulation.
